# Right atrial tuberculoma enclosed by thrombus

**DOI:** 10.21542/gcsp.2024.36

**Published:** 2024-08-01

**Authors:** Freidoun Sabzi, Reza Faraji, Saeed Khoshnood

**Affiliations:** 1 Department of General Surgery, School of Medicine, Kermanshah University of Medical Sciences, Kermanshah, Iran; 2 Tuberculosis and Lung Diseases Research Center, Ilam University of Medical Sciences, Ilam, Iran; 3 Clinical Microbiology Research Center, Ilam University of Medical Sciences, Ilam, Iran

## Abstract

Right atrial tuberculoma (RAT) are an exceedingly rare heart mass often caused by primary pulmonary tuberculosis (TB) in patients infected with human immunodeficiency virus (HIV). In HIV patients, the right atrium is not only a suitable location for tuberculoma formation, but also has a tendency for thrombus formation due to the thrombophilic state of HIV and the destructive effect of the HIV virus on the endothelial surface of the right atrium and subsequent thrombus formation. These masses can simply be detected by transthoracic echocardiography (TEE), but their differentiation from other cardiac pathologies requires histological examination. Herein, using a positive polymerase chain reaction test, we report a case of a previously diagnosed HIV-positive 24-year-old man who presented with a right atrial thrombus, dyspnea, and pleural effusion. Final histological examination identified the right atrial thrombus as a tuberculoma. The patient underwent open heart surgery to remove the mass and received prolonged postoperative TB therapy together with HIV drugs. Three-month follow-up showed some remaining dyspnea and lower extremity edema; however, TEE showed no recurrence of the RAT or thrombus.

## Introduction

Right atrial masses in patients with human immunodeficiency virus (HIV) infection may arise from an HIV-related neoplasm or non-neoplastic process with a wide range of differential diagnosis^1^. The most common reason for right atrial thrombus in these patients is infected thrombus or attachment of vegetation to the tricuspid valve (TV), which is induced by an infected needle or the use of a central venous catheter in hemodialysis patients with a compromised immune system. Other potential causes include thrombophilia, Burkett’s lymphoma, Kaposi sarcoma, rhabdomyoma, fibroma, rhabdomyosarcoma, lipoma, hemangioma, angiosarcoma, osteosarcoma, and cysts. However, exact preoperative detection of RAT may be challenging and could affect the short-term prognosis^2^. This is not the first case of cardiac tuberculoma, especially in an HIV patient. Hajsadeghi et al. described a cardiac tuberculoma in a 34-year-old human immunodeficiency virus (HIV)-infected male^3^. In the present study, we report a case of tuberculoma and thrombus in a patient with HIV treated with surgery and prolonged medical therapy.

## Case presentation

The patient in this study was a 24-year-old man with a history of HIV from two years earlier, presenting with dyspnea, malaise, and fatigue. He also complained of nocturnal cough, pleuritic chest pain, and orthopnea. His medical history included intravenous heroin abuse and repeated hospital admissions for pulmonary infection. On the last admission, he was treated with both heparin and warfarin for three weeks due to a suspected right atrial thrombus. His lower respiratory tract infection was attributed to septic emboli from an infected right atrial mass and was treated with imipenem. The patient had also received zidovudine, lamivudine, and nevirapine during the past two years.

Owing to the viral loads, his last CD4 examination was not available. The patient also had renal failure with proteinuria and increased serum creatinine levels, which were subsequently treated with a short course of oral prednisolone. The patient started crystal and cigarette smoking and alcohol drinking 4 years ago. On admission, he was febrile (temperature, 39 °C) with a low arterial saturation of 83% on room air. His blood pressure was 90/60 mmHg with a pulse rate of 120 beats per minute. There was engorgement of both jugular veins and severe bilateral ankle pitting edema.

Physical examination revealed a systolic murmur in the right 4th intercostal space and coarse crackles on lung auscultation. Electrocardiography demonstrated a normal sinus rhythm of 120 beats per minute, with no evidence of right or left bundle branch block. Chest radiography revealed mild cardiomegaly with patchy infiltration in both lung fields. Laboratory results were as follows: white blood cell count (16  × 109/L), serum hemoglobin (10 g/dl), platelet count (154  × 109/l), sodium (129 mmol/l), potassium (4.9 mmol/l), urea (60 mmol/l), creatinine (3.4 mg/dl), ALP (354 U/l), ALT (56 U/l), AST (65 U/l), total protein (7.5 g/l), and albumin (4.6 g/l). Arterial blood gas analysis revealed a pH of 7.34, PC02 of 45 mmHg, PO2 of 82 mmHg, and HC03 of 25.1 meq/dl. Pericardial fluid examination was positive for polymerase chain reaction (PCR).

**Figure 1.**
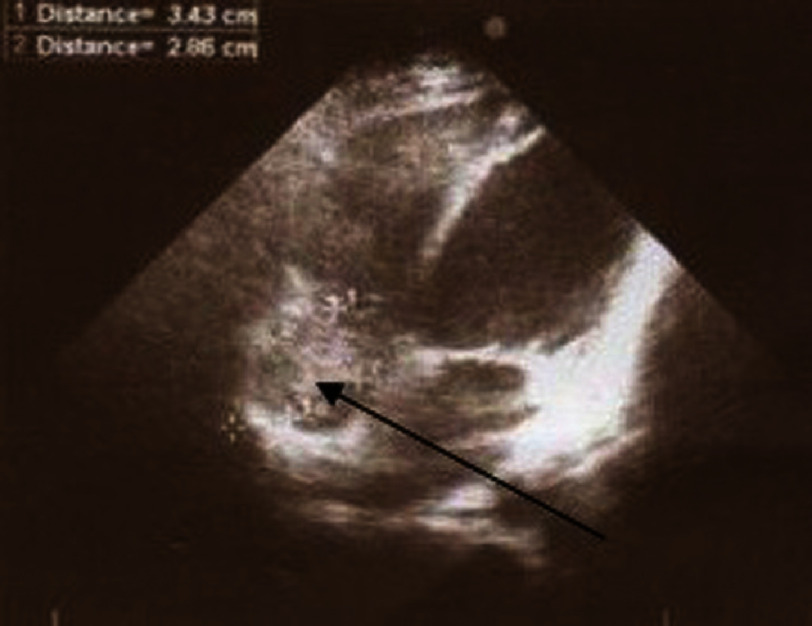
Round mass in right atrium (black arrow).

Two-dimensional transthoracic echocardiography revealed a large, round, non-mobile mass in the right atrium (Figure 1). The clot was attached inferiorly to the interatrial septum and lateral wall of the right atrium, and extended medially to the septal leaflet of the TV. The adhesion of the clot to the TV caused destruction of the septal leaflet and mild-to-moderate TV regurgitation. The right atrium and ventricle were mildly dilated. In addition, the right ventricular systolic function was reduced, but the left ventricular systolic function was normal. Moderate pericardial effusion and no extension of the clot into the inferior or superior vena cava were observed. Despite three weeks of anticoagulant therapy, the size of the suspected clot did not reduce, and he was scheduled for surgery.

**Figure 2.**
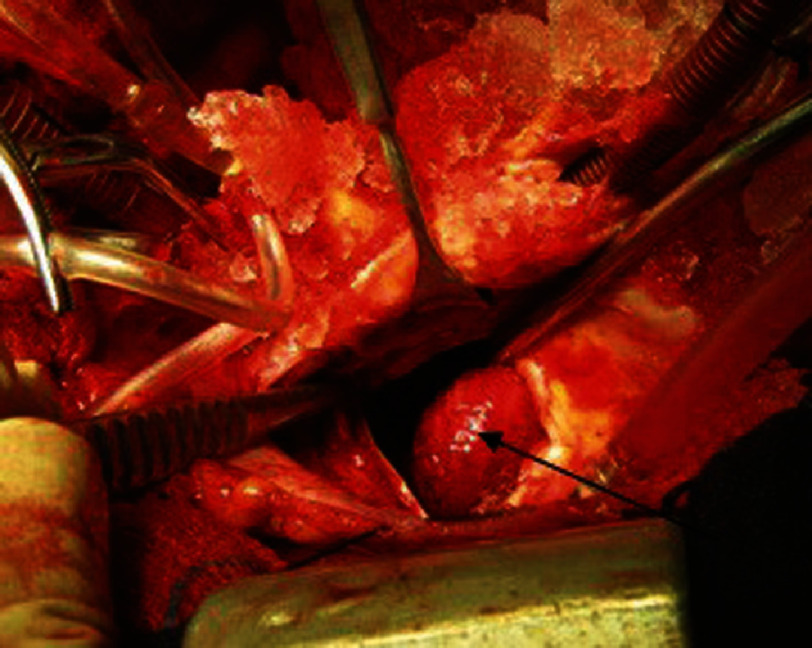
Tuberculoma in right atrium with surrounding fibrin clot (black arrow).

Median sternotomy was performed, and following bicaval and aortic cannulation and encircling tapes around both cava, the right atrium was opened. Intra-operative inspection of right atrium revealed a fragile round creamy colored atrial mass (4  × 4  × three cm) attaching to the interseptal wall and septal tricuspid wall. It was free from inferior vena cava and coronary sinus ostium. The right atrial mass was easily detached from the interatrial septum and completely removed, along with a destroyed septal leaflet (Figure 2).

**Figure 3.**
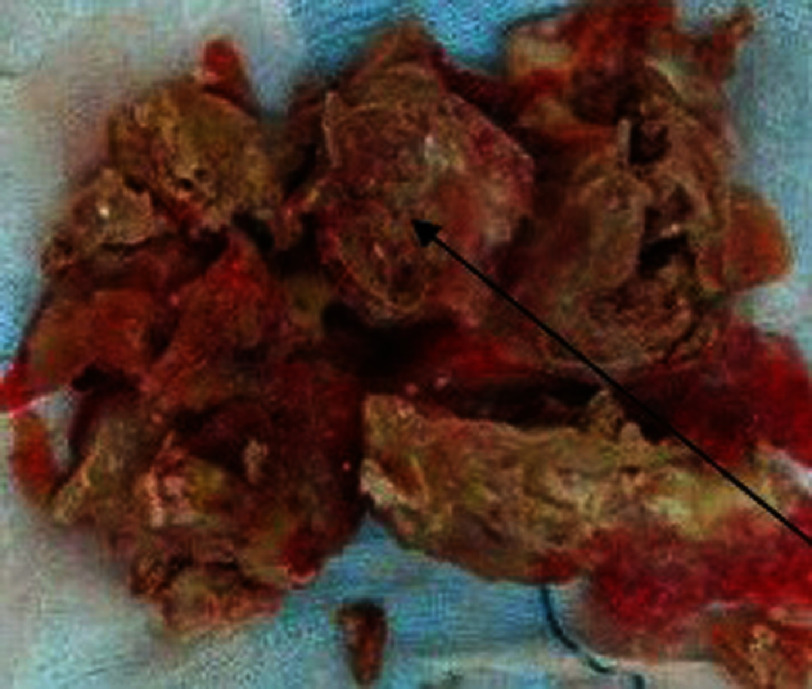
Central caseous necrosis (black arrow) covered by thrombus.

**Figure 4.**
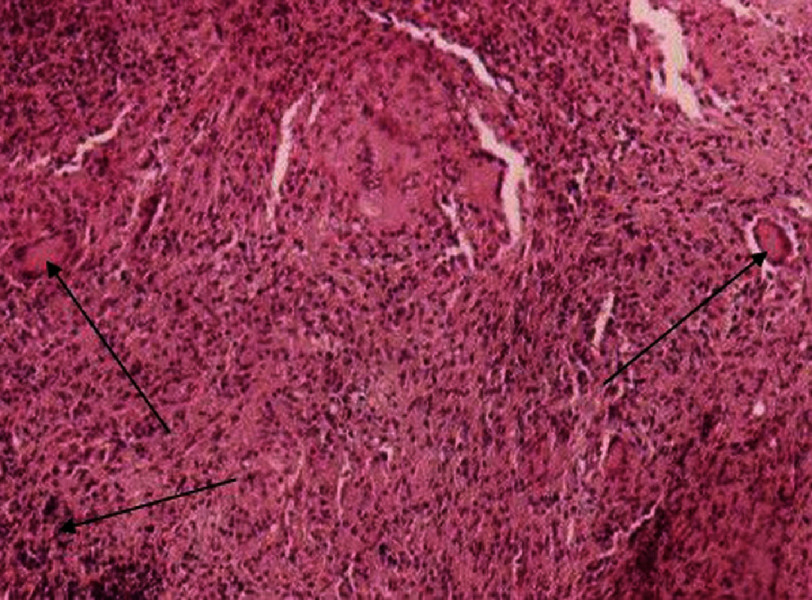
Giant cell of Langhans type (black arrow) in histology of tuberculoma (H&E 100).

**Figure 5.**
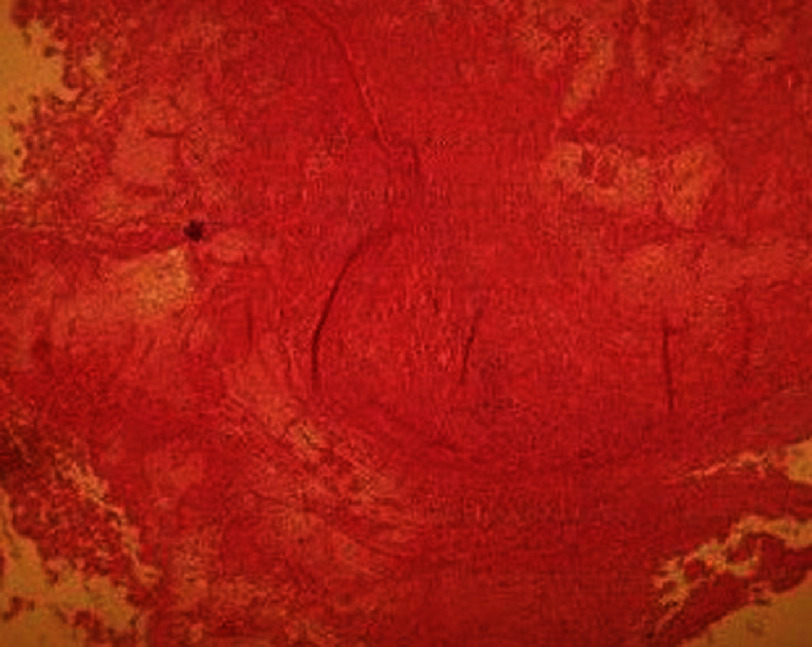
Outer layer of tuberculoma with fibrin strand and red cells (H&E 40).

Histology revealed thrombotic tissue with collar fibrin and red cells and a central nidus of the foreign body reaction of Langhans giant cells. Similar to TB, these Langhans epithelioid cells have been reported in many other pathology studies, and a definite diagnosis was made using a TB-specific test, that is, PCR. The association of positive PCR test with histology finding of aforementioned epithelioid cells, was compatible with a tuberculoma enclosed by thrombus (Figures 3, 4, 5). In addition to excision of the infected mass, the destroyed septal leaflet of the TV was removed. There was also a medium-yellow pericardial effusion, in which the smear and culture were negative for TB or other conventional bacteria. The postoperative course was complicated by a low cardiac output due to right-sided heart failure. The complication was managed by administering inotropic drugs, which were tapered and discontinued on the 5th day of the operation. The patient, while receiving anti-tuberculosis (TB) drugs, was discharged home on the 15th day of the operation. The patient received anti-TB for 12 months including for the first two months four drugs (isoniazid, ethambutol, rifampin and pyrazinamide) and 8 months on a two-drugs regime (isoniazid and rifampin).

## Discussion

TB and HIV have been linked together since the emergence of AIDS 40 years ago. Based on the World Health Organization report, 33% of the world’s population have dormant pulmonary tuberculosis. HIV as a specific immune-compromising disease is likely associated with the activation of these bacilli and their transfer to other organs by systemic circulation^3^.

TB is the most common opportunistic infection and cause of death in HIV patients^4^. The recent global rise in the incidence of TB has contributed to the increased prevalence of HIV^5^. HIV causes a continuous decline in macrophage-mediated immunity, and by altering macrophage defense barrier, leads to the escape of tubercular bacilli from this defense line to circulation, thereby resulting in extra pulmonary TB. The HIV-infected patient with coexisting TB, had an atypical radiographic appearance. The percentage of culture- or smear-positive TB was very low, which may have impeded timely diagnosis of TB.

In the least developed countries, where HIV infection and TB are endemic and health resources are deficient, the prevalence of these diseases continues on an upward slope. The differential diagnosis of a right atrial mass in HIV patients includes endocarditis-related vegetation, malignancy, and thrombus formation^6^. The incidence of cardiac tuberculosis in all TB patients has been estimated to be approximately 0.2–0.3% in post-mortem necropsy. The involvement of cardiac structures without concomitant TB in other organs is a very rare event. The right atrial wall is the most common location of cardiac tuberculomas. The right cardiac chambers, specifically the right atrial free wall, are often involved, possibly because the right mediastinal lymph nodes and lymph chains are directly drained into the right subclavian vein and then into the right atrium^3,5^. They have a well-marked border from the surrounding structures, but adhesion of the tuberculoma to the internal structures of the heart may be associated with injury to the endothelial surface of the cardiac chambers and thrombus formation. Erosion of tuberculoma into the underlying tissue in the interatrial septum leads to conduction deficit. Others rare complication of tuberculoma including, obstruction of inflow or out flow of cardiac chambers, obstruction of superior or inferior vena cava, valve’s erosion and consequent acute regurgitation^4^. Intra-myocardial abscess formation, systemic or pulmonary emboli, and systemic spread of TB to all organ bodies have rarely been reported. Myocardial dysfunction, congestive heart failure, coronary emboli, and sudden cardiac death are late complications of cardiac tuberculoma. Indeed, the prognosis of tuberculoma is related to the size of the mass, the degree of erosion to surrounding structures, and the timely initiation of anti-TB drugs^1,2^.

Most patients with an early diagnosis of cardiac tuberculoma (when the internal texture of the mass is soft and had not any components of calcification), may have their condition resolved by long-term anticoagulant and anti-TB treatment. However, resolution of the tuberculoma in latter stages of the disease is almost impossible when diagnosis is missed and some evidence of calcification is found in the pathological examination.

Although infectious masses are rare, it should be considered in any subjects with intracardiac mass who have had contact with TB^6^. Three specific pathological forms of cardiac TB are diagnosed: the diffuse type, characterized by the presence of giant cells and lymphocytes; the miliary type, characterized by hematogenous spread; and the glandular type, characterized by core caseation.

Ziehl-Neelsen examination of the mass specimens usually fails to shows TB bacilli and a definitive diagnosis depends on a tuberculosis PCR test. The mechanisms underlying tuberculoma formation in these patients have not been completely elucidated. It is unknown how the tubercle bacilli nest over the previously performed thrombus *via* systemic circulation or how the tubercle bacilli captured by the injured endothelial cell surface of the TV cause spotted pin-pointed endothelial cell injury and clot formation^7^. In our case, despite intensive anticoagulant therapy for three weeks, no change in clot size was observed and we decided to opt for surgery.

However, endothelial injury may contribute to the formation of a right atrial thrombus. Intra-myocardial or intraseptal tuberculoma formation can be the result of direct lymphatic extension from pulmonary hilar lymph nodes or retrograde transfer from lymphatic connection between pericardium and myocardium. Right atrial TB in patients with HIV usually originates from hematogenous seeding from a primary pulmonary source. Other predisposing factors for tuberculoma formation in the right atrium include stasis, thrombosis, infected catheter, and intravenous drug abuse, which may predispose to easy nesting of bacilli on the previously performed clot in the right atrium^8,9^.

## Conclusion

HIV is associated with thrombophilia, with a high risk of thrombus formation in intracardiac chambers. Right atrial thrombi may be further complicated by the hematogenous spread of TB bacilli. The lung parenchyma involved in TB may be a primary pathological process or a secondary event due to thromboembolism of the right atrial tuberculoma. This case exhibited a large and mixed granulomatous and thrombotic right atrial mass that was probably caused by hematogenous migration of tubercular bacilli to the right atrium that further by endothelial damage and provides a basis for further accumulation of fibrin on the central nidus of tuberculoma. Surgical removal of an infected right atrial thrombus could serve as a treatment approach for managing right atrial thrombi, especially in patients at high risk of thromboembolism. Cardiac involvement in tuberculosis is associated with an unfavorable prognosis. In this regard, a multidisciplinary cardiologist–pneumonologist–infectious disease specialist approach combined with modern means of risk estimation may be a way to manage these patients effectively.

### What have we learnt?

Right atrial tuberculoma (RAT) are exceedingly rare heart masses that is often caused by primary pulmonary tuberculosis (TB) in human immunodeficiency virus (HIV) patients.

### Funding

This research has not received any specific grant from public, commercial, or non-profit sector agencies.

## Conflicts of Interest

The authors have no conflicts of interest to declare.
